# Effects of Ipratropium Bromide Combined with Traditional Chinese Medicine Intervention on the Pulmonary Function and Psychological Status of Patients with Chronic Obstructive Pulmonary Disease

**DOI:** 10.1155/2023/6483785

**Published:** 2023-02-07

**Authors:** Wen-Yi Ye, Hong Zhao, Jing Ye, Hai-Jin Sang, Lin-Shui Zhou, Jia-Ni Lv, Juan-Juan Li

**Affiliations:** ^1^The First Affiliated Hospital of Zhejiang Chinese Medical University, Hangzhou 310006, China; ^2^The First Affiliated Hospital Zhejiang University School of Medicine, Hangzhou 310003, China; ^3^Tongde Hospital of Zhejiang Province, Hangzhou 310012, China

## Abstract

Recently, most scholars have advocated multidisciplinary comprehensive intervention measures for chronic obstructive pulmonary disease (COPD) to improve lung function, relieve symptoms of dyspnea, and improve quality of life. Traditional Chinese medicine (TCM) has rich experience in the treatment of various respiratory system diseases and the rehabilitation of their syndrome differentiation. In this study, total 68 patients with COPD from November 2019 to November 2021 in the hospitals were divided into the control group, ipratropium bromide (IB)-treated group, and IB + TCM-treated group for clinical efficacy observation and to explore the effect of IB combined with TCM on the pulmonary function and psychological status of COPD patients. Patients in the control group were subjected to routine oxygen inhalation, cough and expectorant, and antiviral treatments, while the patients in the IB-treated group were treated with IB and those received in the control group. Patients in the IB + TCM-treated group were treated with IB and TCM intervention. All patients were treated for a month. The results showed that after different interventions, the levels of FEV1, FEV1% pred, FVC, and PEF (*P* < 0.05) were significantly increased in all the groups, while levels of TNF-*α*, IL-6, IL-8, and CRP in serum as well as Hamilton Anxiety Scale and Hamilton Depression scores were significantly decreased. Compared with the control group and IB-treated group, the IB + TCM-treated group presented the greatest changes on all abovementioned indicators and the lowest total incidence of adverse reactions, indicating the biggest improvement of IB + TCM on the symptoms of COPD patients. Therefore, the combination of IB and TCM intervention effectively improved the pulmonary function and psychological status of COPD patients and could be used as an important adjunct for COPD treatment.

## 1. Introduction

Chronic obstructive pulmonary disease (COPD) is a disease with high morbidity and mortality in the world [1]. And growing evidence has demonstrated that the long-term challenge of COPD significantly increases the risk of lung cancer, chronic pulmonary heart disease, pulmonic encephalopathy, and hypercapnic respiratory failure [2–5]. Especially, as the disease continues, the COPD patients could experience unstable psychological statuses including anxiety, depression, and irritability by the long illness [6, 7]. However, up until now, the pathogenesis of COPD has not yet been well elucidated, but it is believed that cigarette smoke is greatly implicated in the development of COPD [8]. The main feature of the disease is the progressive decline of respiratory function, which seriously affects the quality of life of patients. Routine treatments that are mostly used are decongestants, antihistamines, anticough medicines, expectorants, and antiviral agents which can reduce the mucus secretion in the patient's airway, promote the discharge of sputum, alleviate the patient's clinical symptoms (such as cough and excessive sputum), and reduce secondary infection [9–11]. However, those abovementioned treatments can only play an auxiliary role but have no significant improvement on the patient's lung function, thereby requiring combination therapy for the symptoms [12, 13]. Ipratropium bromide (IB) is a bronchodilator that can directly act on M receptors in the bronchial smooth muscle to inhibit airway remodeling and improve lung function [14, 15].

In recent years, a large number of clinical studies found that traditional Chinese medicine has unique advantages on the treatment of COPD, which can alleviate both the symptoms and the physical states of the patients with COPD [16–18]. The literature showed that the oral traditional decoction and external treatment of traditional Chinese medicine (such as acupoint sticking, acupuncture, and traditional Chinese medicine fumigation) of patients with stable COPD results in obvious clinical efficacy [19–23]. Traditional Chinese medicine (TCM) can be based on acupoint massage and supplemented by traditional Chinese medicine sticking through massaging related acupoints, dredging the meridians, promoting expectoration, and timely treatment of patients' abnormal psychological activities, thereby relieving patients' negative emotions [23–26]. In addition, pulmonary rehabilitation is another hallmark of TCM for COPD treatment [27]. Compared with the traditional community cares, TCM rehabilitation, such as Tai Chi, acupoint massage, and acupuncture, can improve pulmonary function, relieve dyspnea, and strengthen the exercise tolerance of patients, leading to the significant role in enhancing the quality of life and prolonging life [28–30]. However, reports on the clinical efficacies of TCM interventions are few. Therefore, this study aims to investigate the effects of ipratropium bromide combined with traditional Chinese medicine and nursing intervention on pulmonary function and the psychological status of patients with COPD through clinical trial observation.

## 2. Materials and Methods

### 2.1. The Population

In accordance with the random number table method, 68 patients with COPD in the hospitals that the author joined were considered from November 2019 to November 2021 and were divided into three groups: the control (20 cases) group, IB-treated group (24 cases), and IB + TCM-treated (24 cases) group.

The control group was composed of 11 males and 9 females with ages ranging from 49 years to 75 years (average: 64.4 ± 3.8 years) and a course of disease of 2–14 years (average: 6.4 ± 2.0 years). Twelve of the participants in the control group were smokers. On the basis of COPD classification, 6, 8, and 7 cases had grades I, II, and III COPD, respectively.

The IB-treated group was composed of 12 males and 12 females with age ranging from 48 years to 75 years (average: 63.9 ± 4.2 years) and disease duration of 2–12 years (average: 6.8 ± 4.2 years). Fourteen of the participants in the ipratropium bromide intervention group were smokers. On COPD classification, 6, 12, and 6 cases had grades I, II, and III COPD, respectively.

The IB + TCM-treated group comprised 14 males and 10 females with age ranging from 51 years to 75 years (average: 65.5 ± 6.2 years) and disease duration of 2–12 years (average: 7.1 ± 2.6 years). Fifteen of the participants in the combined intervention group were smokers. On the COPD classification, 6, 11, and 7 cases had grades I, II, and III COPD, respectively. There was not any significant difference in the general data among the control group, IB-treated group, and IB + TCM-treated group (*P* > 0.05).

According to the diagnostic methods of COPD [31–33], the inclusion criteria were executed as follows: (1) postbronchodilator forced expiratory volume in 1 s (FEV1) less than 65% and ratio of FEV1 to forced vital capacity less than 0.70; (2) the cigarette smoking history ≥ 5 years; (3) COPD exacerbation in the past 3 months; and (4) having chronic bronchitis. The exclusion criteria were executed as follows: (1) with severe heart, liver, kidney, and other diseases; (2) cirrhosis with edema or ascites; (3) received long-acting nitrate; and (4) could not provide informed consent. This study was approved by the in-hospital medical ethics committee, and all patients signed informed consent. Except for the control group, the patients in the IB + TCM-treated group and IB-treated group were treated with IB aerosol inhalation.

### 2.2. IB and TCM Treatment

Patients in the control group were treated with oxygen inhalation, anticough medicines, expectorants and antiviral agents, and glucocorticoids.

Patients in the IB-treated group were intervened with the treatments of the control group and IB inhalation for adjuvant therapy (two sprays each time for four times a day). Treatment was continued for a month.

Patients in the IB + TCM-treated group were intervened with IB and TCM nursing. Specific intervention methods were emotional intervention, exercise intervention, and acupoint application. (1) Emotional intervention was performed to understand the patient's psychological state, divert the patient's attention through emotional counseling, and avoid major emotional fluctuations. (2) During the exercise intervention, the patient was daily guided to practice Taijiquan for 20 min and walk for 30 min. (3) During acupoint application, 15 g each of fennel, golden cherry, corydalis tuber, and asarum were collected and ground into powder. The powder was then mixed with ginger juice and glycerin to make a medicine cake (height = 25 mm, radius = 6 mm) and applied to Shenshu, Feishu, and Pishu once a day for 1-2 h each time. Treatment was continued for a month.

The randomization was carried out as shown on randomizer.org. The randomization list was generated by an independent researcher who had no further involvement in the conduct of the study and who closely monitored the intervention to ensure that the program ran as expected. Except the independent researcher, all the investigators, collaborators, and study staffs did not know the details of the randomization. The packaging of the test samples and control products was identical in appearance.

### 2.3. Observation Indicators

#### 2.3.1. Pulmonary Function Index and Activity Capacity

The pulmonary function tester was used to measure FEV1, FEV1% pred, peak expiratory flow (PEF), and FVC (forced vital capacity) in all the groups before and after treatment. The BODE score was used to evaluate the patient's body mass index, degree of airflow obstruction, dyspnea, and exercise capacity. The full score was 10 points, and low scores indicated good patient mobility.

#### 2.3.2. Inflammation-Related Mediator Levels

About 5 mL fasting venous blood was collected from the two groups of patients and centrifuged at 3000 r/min for 15 min. The upper serum was collected to detect the levels of tumor necrosis factor-*α* (TNF-*α*), interleukin-6 (IL-6), interleukin-8 (IL-8), and C reactive protein (CRP) in serum by using enzyme-linked immunosorbent assay.

#### 2.3.3. Mental Condition

The Hamilton Anxiety Scale (HAMA) and the Hamilton Depression Scale (HAMD) were used to evaluate the psychological status of the two groups of patients before and after treatment. Among them, the score range for HAMA was 0–56. A high score indicated severe anxiety. HAMD adopted the version with 17 items. The total score was 68 points, and a high score indicated severe depression.

#### 2.3.4. Adverse Reactions

The occurrence of adverse reactions during treatment, including spontaneous pneumothorax, chronic respiratory failure, chronic pulmonary heart disease, and sleep disordered breathing, in the three groups was compared.

### 2.4. Statistical Analysis

In this study, the SPSS 22.0 statistical software was used to analyze the data. The data were expressed as the mean ± SD. For normal distribution, chi-square tests and a two-sided Student's *t* test were used for baseline comparisons of the IB-treated and IB + TCM-treated groups regarding continuous and categorical variables, respectively. The highly skewed continuous and categorical variables were compared using the Wilcoxon signed-rank sum tests and Fisher's exact test, respectively. *P* < 0.05 indicated statistically significant difference.

## 3. Results

### 3.1. Effects of IB and TCM on the Pulmonary Function Index and Activity Capacity

As shown in Table 1, before intervention, there were not any significant differences on the levels of FEV1, FEV1% pred, FVC, and PEF among the 3 groups, indicating the same pulmonary function index and activity capacity of the patients in this study. However, after one month of intervention, the levels of FEV1, FEV1% pred, FVC, and PEF of each group were significantly increased in some degrees (*P* < 0.05) compared with those in the control group before intervention, while the BODE index was significantly decreased (*P* < 0.05). Moreover, the levels of FEV1, FEV1% pred, FVC, and PEF in the IB + TCM-treated group were much higher than those in the IB-treated group (*P* < 0.05), indicating that IB and TCM combination could present better outcomes in COPD patients.

### 3.2. Effects of IB and TCM on the Levels of Inflammatory Mediators in Serum

As shown in Table 2, compared to before intervention, the levels of serum TNF-*α*, IL-6, IL-8, and CRP in each group were significantly decreased after different treatments. Especially, the serum levels of those inflammatory cytokines in the IB + TCM-treated group were significantly reduced compared with those in the IB-treated group (*P* < 0.05), indicating that the combination of IB and TCM was significantly produced more inhibition on inflammatory response in vivo than individual IB alone.

### 3.3. Effects of IB and TCM on Mental Conditions and Adverse Reactions

After a one-month routine intervention in COPD patients, both the HAMA and HAMD scores in the control groups did not decreased compared with those before treatment. However, both IB treatment alone and combination of IB and TCM for one month could significantly improve the mental conditions of the patients (*P* < 0.05), as shown in Table 3. During the treatment periods, although there was no significant difference observed in the total incidence of adverse reactions among the control group, the IB-treated group, and the IB + TCM-treated group (*P* > 0.05), the total incidence of adverse reactions in the IB-treated group and IB + TCM-treated group was much lower than that in the control group (Table 4).

## 4. Discussion

COPD is a common and preventable respiratory disease characterized by persistent airflow limitation. Except *β*2 agonist and glucocorticoid, growing novel strategies, such as Nrf2 agonist, prebiotics, stem cell transplantation, and oligonucleotides have been considered [34–40], but there is no efficient treatment to prevent its progression. Patients are treated with drugs to keep the airway unobstructed and improve the symptoms of dyspnea, chest tightness, and wheezing [13]. However, conventional treatments, such as oxygen inhalation, phlegm removal, and anti-infection, can only relieve the symptoms of the patient's disease and fail to reach lesions. Moreover, the control effect of COPD is closely related to the psychological state of the patient [41]. Repeated episodes of the disease can lead to adverse psychological conditions in patients, such as anxiety and depression, which further aggravate the disease and make patients physically and mentally distressed [41, 42]. Therefore, appropriate psychological nursing interventions should be supplemented during treatment.

Traditional Chinese medicine has unique advantages in the treatment of COPD. Clinical practice has confirmed that traditional Chinese medicine nursing has a good effect on relieving symptoms, such as shortness of breath and dyspnea, in patients with chronic respiratory diseases [32, 43–49]. In this study, patients with COPD were treated with TCM nursing measures, such as emotional nursing, exercise intervention, and acupoint massage, in accordance with the principles of syndrome differentiation and nursing. Results showed that observation groups had higher predicted values of FEV1/FVC and FEV1, as well as lower dyspnea and CAT scores than the control group. TCM nursing is suggested to improve the lung function of patients with COPD to a certain extent, relieve the symptoms of dyspnea, and improve the quality of life. Chinese medicine pays attention to the holism of the physiological functions of the human body and believes that the various components including immune cells, epithelial cells, vascular endothelial cells, and neurocytes, in the human body are coordinated and affect each other, mutually contributing to the development of COPD [50, 51]. Patients with COPD often have difficulty in breathing due to decreased lung function, which leads to decreased mobility and may lead to depression and other negative emotions [52]. TCM nursing uses emotional care, such as emotional counseling, to adjust the abnormal changes in patients' emotions. Therefore, patients can be unified physically and mentally. In addition, acupoint application and massage are beneficial to promote the metabolism and blood circulation of the patients, improve the symptoms, lung function, and blood-gas indicators, and enhance the quality of life, and more importantly, they are stable and reliable without evident side effects [53, 54]. In this study, compared with the IB-treated group, the patients intervened with IB and TCM nursing had better mental status and lower incidence of adverse reactions. It indicated that TCM nursing could be used as an important adjunctive treatment for COPD.

IB is a highly selective M3 subtype cholinergic receptor blocker [55]. After administration, ipratropium bromide can act on the M receptor of the bronchial smooth muscle, mediate the neurotransmitter biological effects of sympathetic and parasympathetic nerves, block acetylcholine, dilate bronchi, alleviate bronchospasm, and improve lung function [56]. Intensive care for psychological stress not only can provide a relatively overall understanding of the psychology of the patient through clinical diagnosis but also can obtain the reasons affecting the patient's mood according to his personality and family relationships over time [57, 58]. Moreover, according to the psychological problems of different patients, individualized cares can be developed to resolve their negative emotions and provide favorable conditions for improvement of their condition as well as increase their mobility due to negative emotions [59, 60]. The results of this study showed that after IB or IB + TCM-combined treatment, the patients in these two groups had much higher FEV1, FEV1% pred, PEF, FVC, and BODE indicators but lower HAMA and HAMD scores than those in the control group. It suggested that treatment with IB alone and cotreatment with IB and TCM nursing, could effectively improve lung function, activity ability, and psychological status of the patients with COPD.

The levels of inflammatory mediators, such as TNF-*α*, IL-6, IL-8, IL-17, and CRP, in the serum of COPD patients were highly expressed, and the lung structure was damaged due to the increased levels of inflammatory cytokines, resulting in the accumulation of a large number of T lymphocytes and neutrophils in the respiratory mucosa and causing airway reactions [61–63]. IB adjuvant therapy can directly act on the inflammatory site through airway inhalation and reduce the secretion of respiratory mucosa and the accumulation of inflammatory cells, thereby alleviating the inflammatory response [64]. In the study, the results displayed that the serum levels of TNF-*α*, IL-6, IL-8, and CRP in the IB-treated or IB + TCM-cotreated group were significantly lower than those in the control group. The incidences of adverse reactions in the IB- and IB + TCM-treated groups were also much lower than that in the control group. However, due to the small sample size and use of a single center in this study, the overall incidence of adverse reactions in the three groups (control group, IB-treated group, and IB + TCM-treated group) did not present any statistically significant difference. This result suggested that IB combined with TCM nursing as an effective adjuvant treatment for COPD could significantly reduce the levels of inflammatory mediators.

In conclusion, the treatment with IB combined with TCM nursing can effectively enhance lung function, reduce inflammatory response, and improve psychological status of the COPD patients, resulting in the recovery of the disease. TCM nursing combined with IB could be effective in clinical treatment of COPD.

## Figures and Tables

**Table 1:** Effects of ipratropium bromide and combined intervention on the lung function of COPD patients.

Group	Cases (*n*)	FEV_1_ (L)		FEV_1_% pred (%)		FVC (L)		PEF (L/s)		BODE index	
		BT	AT	BT	AT	BT	AT	BT	AT	BT	AT
Control	20	1.35 ± 0.22	1.42 ± 0.14^f^	51.0 ± 4.0	54.1 ± 3.2^cf^	2.58 ± 0.20	2.68 ± 0.22^f^	7.63 ± 0.55	7.58 ± 0.48^f^	6.52 ± 0.58	5.90 ± 0.55^cf^
Ipratropium bromide	24	1.31 ± 0.27	1.65 ± 0.21^ad^	51.3 ± 4.7	58.8 ± 2.9^ade^	2.54 ± 0.18	1.96 ± 0.20^adf^	7.64 ± 0.57	4.41 ± 0.35^ade^	6.49 ± 0.61	4.65 ± 0.43^ade^
Combined intervention	24	1.33 ± 0.21	1.76 ± 0.16^bd^	52.7 ± 4.3	63.5 ± 3.3^bd^	2.57 ± 0.19	1.60 ± 0.15^bd^	7.70 ± 0.63	4.15 ± 0.33^bd^	6.50 ± 0.55	4.15 ± 0.36^bd^

BT, before treatment; AT, after treatment; compared with the control group, ^a^*P* < 0.05, ^b^*P* < 0.01; compared with itself before treatment, ^c^*P* < 0.05, ^d^*P* < 0.01; compared with the ipratropium bromide group, ^e^*P* < 0.05, ^f^*P* < 0.01.

**Table 2:** Effects of ipratropium bromide and combined intervention on levels of proinflammatory cytokines in serum COPD patients.

Group	Cases (*n*)	TNF-*α* (ng/L)		IL-6 (ng/L)		IL-8 (ng/L)		CRP (mg/L)	
		BT	AT	BT	AT	BT	AT	BT	AT
Control	20	35.7 ± 5.32	31.1 ± 5.71^cf^	57.2 ± 6.30	60.7 ± 5.63^f^	24.8 ± 2.55	23.1 ± 1.94^f^	24.2 ± 2.65	21.9 ± 3.16^cf^
Ipratropium bromide	24	35.1 ± 5.28	16.7 ± 2.25^adf^	56.9 ± 5.48	39.3 ± 4.79^adf^	24.7 ± 3.05	10.8 ± 0.96^adf^	23.9 ± 2.13	16.4 ± 1.22^adf^
Combined intervention	24	36.05 ± 5.36	13.5 ± 1.16^bd^	56.6 ± 5.12	30.7 ± 2.36^bd^	24.3 ± 2.61	8.65 ± 0.66^bd^	24.6 ± 2.25	12.4 ± 2.10^bd^

BT, before treatment; AT, after treatment; compared with the control group, ^a^*P* < 0.05, ^b^*P* < 0.01; compared with itself before treatment, ^c^*P* < 0.05, ^d^*P* < 0.01; compared with the ipratropium bromide group, ^f^*P* < 0.01.

**Table 3:** Effects of ipratropium bromide and combined intervention on mental condition of COPD patients.

Group	Cases (*n*)	HAMA		HAMD	
		BT	AT	BT	AT
Control	20	15.6 ± 4.24	15.3 ± 3.24^f^	14.6 ± 3.40	13.3 ± 2.82^cf^
Ipratropium bromide	24	16.0 ± 4.57	7.65 ± 3.50^ad^	14.9 ± 4.15	8.64 ± 2.36^ad^
Combined intervention	24	15.4 ± 4.15	6.37 ± 2.15^bd^	13.9 ± 3.25	8.12 ± 2.06^ad^

BT, before treatment; AT, after treatment; compared with the control group, ^a^*P* < 0.05, ^b^*P* < 0.01; compared with itself before treatment, ^c^*P* < 0.05, ^d^*P* < 0.01; compared with the ipratropium bromide group, ^f^*P* < 0.01.

**Table 4:** Adverse reactions in the ipratropium bromide group and combined intervention group.

Group	Cases (*n*)	Spontaneous pneumothorax	Chronic respiratory failure	Chronic pulmonary heart disease	Sleep apnea	Total incidence
Control	20	2	2	1	1	6^f^
Ipratropium bromide	24	1	2	1	0	4
Combined intervention	24	1	1	1	0	3^a^

BT, before treatment; AT, after treatment; compared with the control group, ^a^*P* < 0.05, compared with the ipratropium bromide group, ^f^*P* < 0.01.

## Data Availability

The data used to support the findings are available from the corresponding authors upon reasonable request.
